# Primary Gastrointestinal Stromal Tumor of the Prostate: Unexpected Guest

**DOI:** 10.7759/cureus.10244

**Published:** 2020-09-04

**Authors:** Haneen Al-Maghrabi, Shadi Alahmadi, Afnan H Falemban

**Affiliations:** 1 Pathology, King Faisal Specialist Hospital and Research Centre, Jeddah, SAU; 2 Pathology, King Abdulaziz University, Jeddah, SAU; 3 Pathology, Al-Noor Specialist Hospital, Makkah, SAU

**Keywords:** gist, prostate, needle biopsy, gleevec, imatinib, cd117, c-kit, prostatic, gastrointestinal stromal tumor, kit

## Abstract

Primary mesenchymal lesions of the prostate are exceptionally rare. They comprise 1% of all prostatic neoplasms. Despite its rare location, the diagnosis of primary gastrointestinal stromal tumors (GISTs) of the prostate gland should never be missed. Such a diagnosis can be made after the rolling out of direct extension from adjacent organs, especially the rectum. GIST diagnosis has a clinical impact on patient treatment and clinical outcomes. They harbor a certain KIT activation mutation that responds to pharmacologic therapy inhibitors.

The objective of the current study was to provide a thorough review of GIST arising primarily in the prostate gland along with a comprehensive study of GIST pathogenesis, histologic morphology, immunohistochemistry, and molecular studies’ findings, and their importance in differentiating GIST from other prostate mesenchymal tumors. This will emphasize the role of careful spindle cell lesion diagnosis in the prostate gland that can influence the prognostic stratification of clinical management, future follow-up, and disease outcome.

Thirteen cases were collected after an extensive and detailed review of the English literature through PubMed, Medknow, Google Scholar, as well as personal experience.

The anatomic location of this lesion plays a significant role in the differential diagnosis. It is difficult to establish the accurate primary origin of GIST on core needle tissue biopsy. Thus, clinical, and radiological examinations play a crucial role in rolling out the possibility of rectal GIST secondarily invading and involving the prostate gland.

To conclude, primary prostatic GIST is a rare diagnosis. Extraintestinal, particularly rectal, GIST can clinically and radiologically mimic the impression of the prostatic lesion. Before diagnosing primary prostatic spindle cell lesions, such as solitary fibrous tumor (SFT), inflammatory myofibroblastic tumor (IMT), leiomyoma, leiomyosarcoma, or prostatic stromal tumors, one should include CD117/c-Kit in the workup of a prostatic spindle cell lesion. GIST has distinct pathogenesis, and its diagnosis can have a clinical impact on the patient's management plan and clinical outcome.

## Introduction and background

Gastrointestinal stromal neoplasms (GISTs) are well-known mesenchymal tumors that commonly arise in the gastrointestinal (GI) tract. The stomach, small bowel, and rectum are the most frequently involved anatomic locations. Interstitial cells of Cajal are considered the bioelectrical pacemaker cells of gut motility and display GIST differentiation. Interestingly, GISTs have been reported outside the GI tract in areas that lack the cells of Cajal such as bowel mesentery, omentum, and retroperitoneum [1]. CD117 (c-KIT) is expressed in most interstitial cells of Cajal and became a specific marker to diagnose GIST. At the molecular level, GISTs harbor an activating KIT mutation from a platelet-derived growth factor receptor alpha (PDGF-a), receptor tyrosine kinase, which makes them suitable for certain inhibitors such as imatinib mesylate and sunitinib malate [2]. Therefore, it is crucial for patient management to distinguish primary prostatic GIST from other mesenchymal spindle cell lesions, both clinically and histologically. Spindle cell lesions of the prostate gland involve a wide differential diagnosis due to a lack of specific clinical signs and symptoms, laboratory results, low reported incidence, and unexpected anatomic location. The correct final pathologic diagnosis of a spindle cell lesion on a core biopsy can be difficult to reach. This could be due to morphology overlapping between mesenchymal and/or epithelial tumors, lack of total tumor representation through limited needle biopsy sample, and availability of remaining tissue in the paraffin block for necessary ancillary studies. A primary spindle cell lesion of the prostate includes prostatic stromal tumors (prostatic stromal tumor of uncertain malignant potential, prostatic stromal sarcoma), leiomyoma, hemangioma, neurofibroma, leiomyosarcoma, rhabdomyosarcoma, and direct extension of mesenchymal tumors from adjacent organs, such as GIST, of the gastrointestinal tract, especially the rectum [3]. The latter group should be carefully ruled out before diagnosing primary prostatic GIST. In our study, we reviewed previously published cases of GIST arising primarily in the prostatic gland along with detailed differential diagnosis and their histologic examination results. Also, a summary of the clinical, radiological, and histopathology findings of reported cases is provided.

## Review

Materials and methods

Thirteen cases were collected after an extensive review of the English literature from PubMed, Medknow, and Google Scholar. All these published cases were examined by paraffin-embedded hematoxylin and eosin-stained (H&E) sections. Immunohistochemical staining studies were carried out among all of them to confirm the diagnosis of GIST. This included cluster of differentiation 117 or CD117 (all 13 cases), CD34 (12 cases), and DOG1 (5 cases). We followed focused and selective research on primary prostatic GIST cases. The inclusion criteria of our study were limited to tumors originating primarily in the prostate gland and confirmed by clinical and radiological studies. Cases that were suspicious, suggestive, contiguous, or raised between the rectum and adjacent organs were excluded from our search.

Result

GISTs of the prostate are extremely rare. Only a few case reports of primary GIST in prostate glands have been reported. The anatomic location of this lesion plays a significant role in the differential diagnosis. It is difficult to establish the accurate primary origin of a GIST on core needle tissue biopsy. Thus, clinical and radiological examinations play a crucial role in rolling out the possibility of rectal GIST secondarily invading and involving the prostate gland. However, sometimes, clinical and radiology examinations cannot confirm the primary origin of the tumor. GISTs can arise as a small intramural nodule or as a large pelvic mass with a prostatic extension mimicking primary prostatic GIST. Most of the published studies reported six cases of primary prostatic GIST [4-9]. However, deep digging revealed more cases [10-16]; all are listed in Table 1. Cases that were excluded from our study were extraperitoneal, rectovesical, and retroprostatic masses [17] and masses between the rectum and prostate [18] or contiguous to rectal wall [19].

**Table 1:** Summary of reported cases of primary prostatic E-GIST (n=13) E-GIST: Extra-gastrointestinal stromal tumor; NED: No evidence of disease; DUD: Dead of unrelated disease; DRD: Dead related to disease, NA: not available, TURP: Transurethral resection of the prostate

Reference	Age	Prostate size (cm)	Mitotic count per 50 HPF	Hospital course	Follow-up (months)	Outcomes	Recurrence	Metastasis	Management after recurrence
Almagharbi et al. (2018) [4]	84	17 in aggregate	3	Transvesical open prostatectomy	NA	NA	NA	NA	NA
Liu et al. (2014) [5]	55	10.5	8	Preoperative transperineal biopsy + Radical prostatectomy + oral administration of 400 mg/day of imatinib (IM)	12	NED	No	No	None
Zhang et al. (2014) [6]	31	6.5	More than 10	TRUS guided prostate biopsy + no surgery + imatinib (400 mg per day)	3	DUD (electrolyte disturbances and multiple organ failure).	mass volume increased (6.5 × 7.2 × 9.0 cm)	No	None
Yinghao et al. (2007) [7]	49	8.5	More than 5	Radical prostatectomy	14	NED	No	No	None
Lee et al. (2006) [8]	75	6.7	15	TURP + Radical prostatectomy	6	NED	No	No	None
Van der Aa et al. (2005) [9]	49	14.2	Abundant mitosis	Transperineal biopsy + imatinib mesylate	25	Reduced mass volume and liver nodules	NA	Multiple liver metastases (at first time diagnosis)	None
Schöffski et al. (2019) [10] ‏	60	12 (at first time diagnosis)	Low (Ki67 1%) – in both times of diagnosis	TURP + followed up the patient without specific intervention (at first time diagnosis). Radical, retro-pubic prostatectomy followed by adjuvant imatinib treatment (after 5 years of primary diagnosis).	36	Tumor shrunk to 7.3 cm in diameter after neoadjuvant treatment, NED	Tumor increased in size, 14 cm (after 5 years from first-time diagnosis)	No	None
You et al. (2018) [11]	66	8 (origin in prostate & invaded anterior rectal wall)	More than 5	Exploratory laparotomy + radical prostatectomy + rectum repair + sigmoid colostomy. No adjuvant therapy given.	36	NED	NA	No	None
Huh et al. (2014) [12] ‏	50	11	More than 5	TURP + patient refused radical prostatectomy and did not show for follow up	NA	NA	NA	No	NA
Pamu et al. (2013) [13]	75	6.2	Fewer than 5	TURP + Radical retropubic prostatectomy	6	NED	No	No	None
Ou et al. (2013) [14]	39	10	NA	TURP + radical prostatectomy, followed by targeted therapy with imatinib (400 mg, daily) for 1 year	24	NED	No	No	None
de Carvalho et al. (2010) [15] ‏	92	NA	Abundant mitosis	Retropubic prostatectomy for complete resection only	60	DUD	No	No	None
Park et al. (2008) [16] ‏	58	7.5	Fewer than 5	Suprapubic open prostatectomy + additional retropubic radical prostatectomy again after 2 weeks	6	NED	No	No	None

Clinical features

Mean patient age at the time of diagnosis was 60 years (range from 31 to 92 years old). Most of these patients have a similar nonspecific clinical presentation such as vague perineum pain (n=1), retropubic pain, tenesmus (n=1), feelings of incomplete defecation (n=1), constipation (n=2), bloody stool (n=1), urinary symptoms like frequency, urgency, dysuria, acute urinary retention (n=8), and hematuria (n=2), and abnormal digital rectal examination in all of them. The tumor sizes were variable, ranging from 17 cm in aggregates to 6.2 cm. Serum prostate-specific antigen (PSA) level was reported to be less than 3 ng/ml, ranging from 0.2 to 2.45 ng/ml.

Radiographic features

Radiology findings are always variable depending on the tumor size and anatomical location at presentation. Eight out of 13 patients underwent preoperative transurethral resection biopsy of the prostate (TURP). Clinical rectal examination and TURP biopsy are very useful clinical tools. However, they are not accurate to determine the primary origin of the lesion. A computed tomography (CT) scan, along with magnetic resonance imaging (MRI), are very good tools to visualize the primary origin of the tumor. A CT scan usually reveals a solid heterogeneous mass that reflects the presence of tumor hemorrhage or cystic degeneration. It is also useful to follow up for post-therapy recurrence and or/ metastasis. Ninety percent of the presented cases demonstrate a prostatic mass compressing the rectum and urinary tract system but restricted to the organ’s capsule. Due to the mass compression effect, patients presented mainly with urinary symptoms and bowel discomfort.

Histopathology

**Figure 1:**
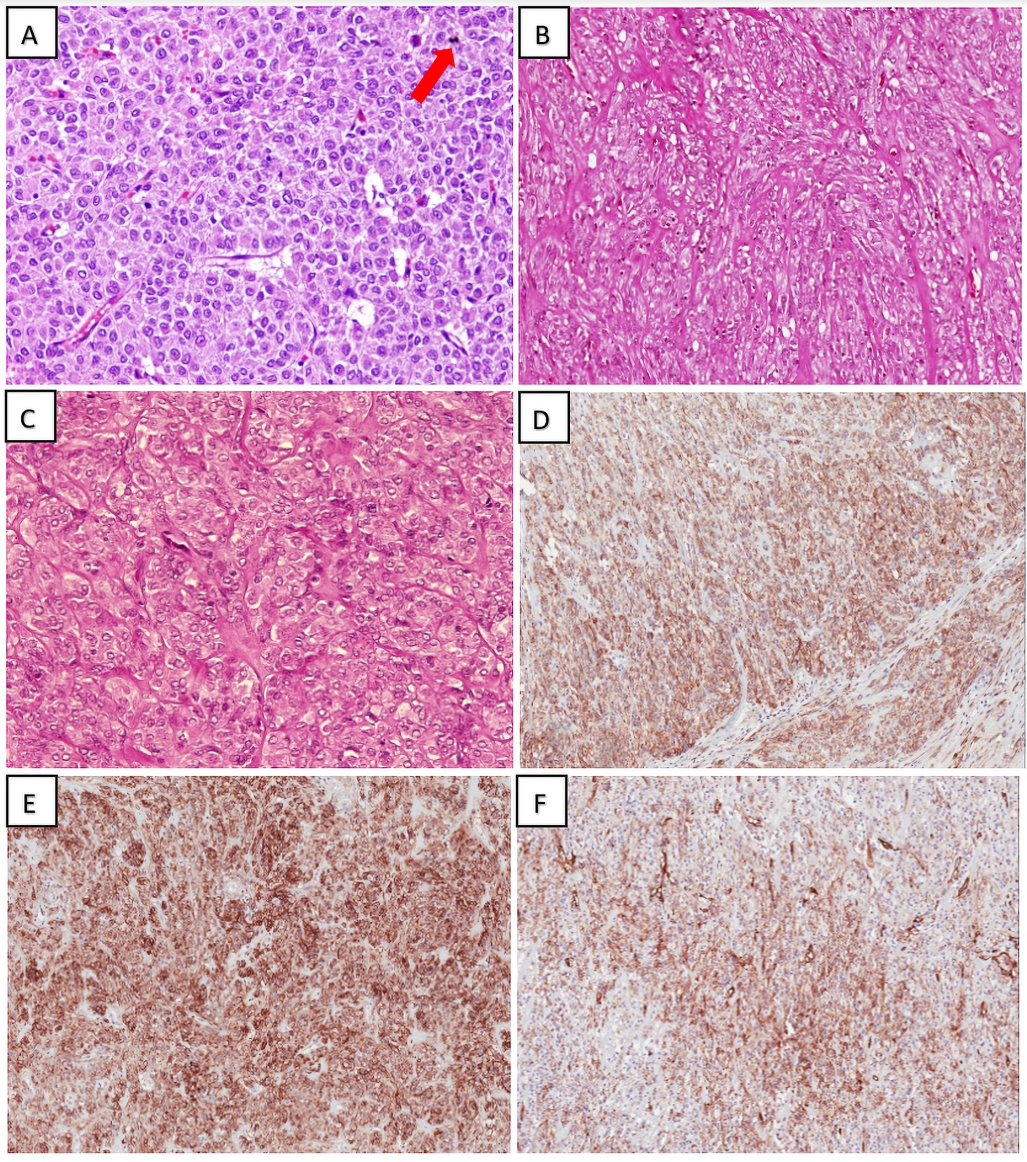
Histopathology of E-GIST in H&E stain and their diagnostic Immunohistochemistry (A) High-power view of epithelioid pattern in E-GIST; note the presence of mitotic figure (red arrow) (H&E; 40x); (B) Spindle cell variant E-GIST with stromal fibrosis and hyalinization (H&E; 40x); (C) Epithelioid pattern in E-GIST demonstrating evenly dispersed, round regular cells arranged in nests (H&E; 40x); (D) Diffuse CD117/c-KIT immunoreactivity within E-GIST (20x); (E) Diffuse and strong DOG1 immune positivity within E-GIST (40x); (F) CD34 expression (fewer tumor cells as compared to CD117 and DOG1) (20x) E-GIST: Extra-gastrointestinal stromal tumor; H&E: hematoxylin and eosin; CD: Cluster of differentiation

Three morphologic variants are identified in GISTs: spindle cells (70%), epithelioid (20%), and mixed morphology (10%) [20]. Spindle cell GISTs are the most common morphology arranged in a syncytial pattern, composed of bland spindle cells, faint eosinophilic cytoplasm, elongated nuclei, and inconspicuous nucleoli. Artifactual para-nuclear vacuoles are commonly seen in gastric GIST. These spindle cell tumors can be seen with a sclerotic background, palisaded cells, vacuolated artifacts, hypercellularity, or sarcomatoid features. Epithelioid GISTs are composed of round cells with clear to eosinophilic cytoplasm arranged in sheets and nests. This histologic variant has more tendency for pleomorphism than the spindle cell type. Mixed GISTs are tumor cells with features between the spindle and epithelioid morphology. The concept of tumor risk assessment is very important for the potential assessment of biological GISTs. The criteria of risk stratification include tumor size and mitosis per 5 mm^2^ or 50 high power field (HPF). These pathologic findings are correlated clinically to the risk of disease progression (which is defined as metastasis or tumor-related death). All reported cases of our study were composed of conventional spindle cell morphology with variable mitotic figures (range from 3 to more than 15/50 HPF). Those patients with high mitosis required more than surgical intervention, including treatment with imatinib target therapy, along with future follow-up. Figure 1 demonstrates the histopathology of E-GIST in hematoxylin and eosin (H&E) stain and their diagnostic immunohistochemistry.

Immunohistochemistry

It is necessary to distinguish any spindle cell lesions from GIST due to the advantage of kinase inhibitor therapy used in GIST treatment. Leiomyoma, schwannoma, desmoid fibromatosis, and leiomyosarcoma can mimic GIST, especially on preoperative core needle tissue. Thus, immunohistochemistry plays a great role to confirm the final diagnosis of GIST. CD117 (c-KIT) must be included in the panel of prostate spindle cell lesions, which stain more than 95% of GISTs. KIT-negative GISTs are epithelioid cells of either gastric or E-GIST origin that harbor PDGF - a mutation. CD117 immunoreactivity is almost always diffuse and strong among all tumor cells, however, rare cases can express focal or patchy staining. Perinuclear dot-like or membranous staining can be seen in tumor cells. CD34 is positive in up to 70%, smooth muscle actin (SMA) expressed in 30%-40%, S100 and desmin can be focally positive in 5%, and cytokeratin is positive focally and weak in 1%-2% of cases [21]. Table 2 summarizes the most practical immunohistochemistry used in the differential diagnosis of prostatic GIST.

**Table 2:** Immunohistochemistry markers useful in differential diagnosis of prostatic GIST GIST: Gastrointestinal stromal tumor, STUMP: Stromal tumor of uncertain malignant potential, SFT: Solitary fibrous tumor, IMT: Inflammatory myofibroblastic tumor

	CK and EMA	S100	SMA	Desmin	CD34	CD117	DOG1	STAT6	Others
GIST	-	-	- / +	-	+	+	+	-	
STUMP	-	-	- / +	+ / -	+	-	-	-	PR+, ER-
Prostatic stromal sarcoma	-	-	-	-	+	-	-	-	
Smooth muscle tumors	- /rare focally +	-	+	+	-	-	-	-	Caldesmon +
SFT	-	-	-	-	+	-	-	+	GRIA2+
IMT	- /rare +	-	+	+ / -	-	-	-	-	ALK+ (2/3 cases), subset ROS1 +
Synovial sarcoma	Focal +	+ / -	-	-	Always -	-	-	-	TLE1+, SOX10+ (5%)

Molecular analysis

**Figure 2:**
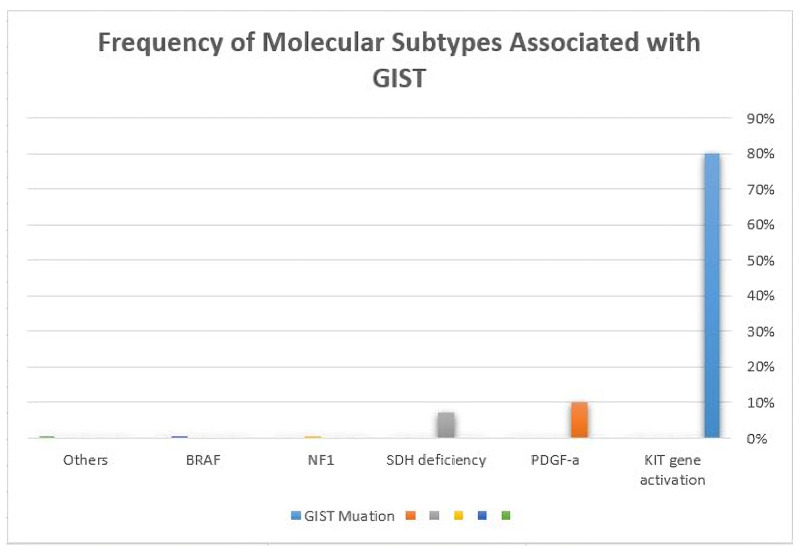
Frequency of molecular subtypes associated with GIST GIST: Gastrointestinal stromal tumor

KIT gene activating mutation occurs in 85% of GISTs; 10% of tumors have the PDGF-a activating mutation gene. ligand-independent activation is activated as a result of KIT protein mutation. The genomic locations and frequency of activating KIT and PDGF-a mutations are variable. Exons 21 contained both KIT and PDGF-a proteins. Exons 9, 11, 13, and 17 are considered the hot spots for KIT mutation. PDGF-a mutations are found in exons 12, 14, and 18. Some GISTs (5-10%) can be negative for both KIT and PDGF-a mutations. Those rare subtypes can harbor other molecular alterations such as the following: Succinate dehydrogenase (SDH)-deficient 7%-13% (SDHA, SDHB, SDHC, and SDHD) and other rare mutations identified as NF1 (0.5%) and BRAF (0.5%) (Figure 2) [22]. For KIT and PDGF-a molecular mutations, analysis is strongly encouraged to be performed in cases with failure to imatinib mesylate therapy response, incomplete tumor resection, or metastasis especially for patients diagnosed with a high-risk group pathology. It must be known that secondary acquired mutations in exons 13, 14, and 17 are usually detected as point mutation after tumor therapy resistance or after a long-term imatinib course [23].

Management

**Figure 3:**
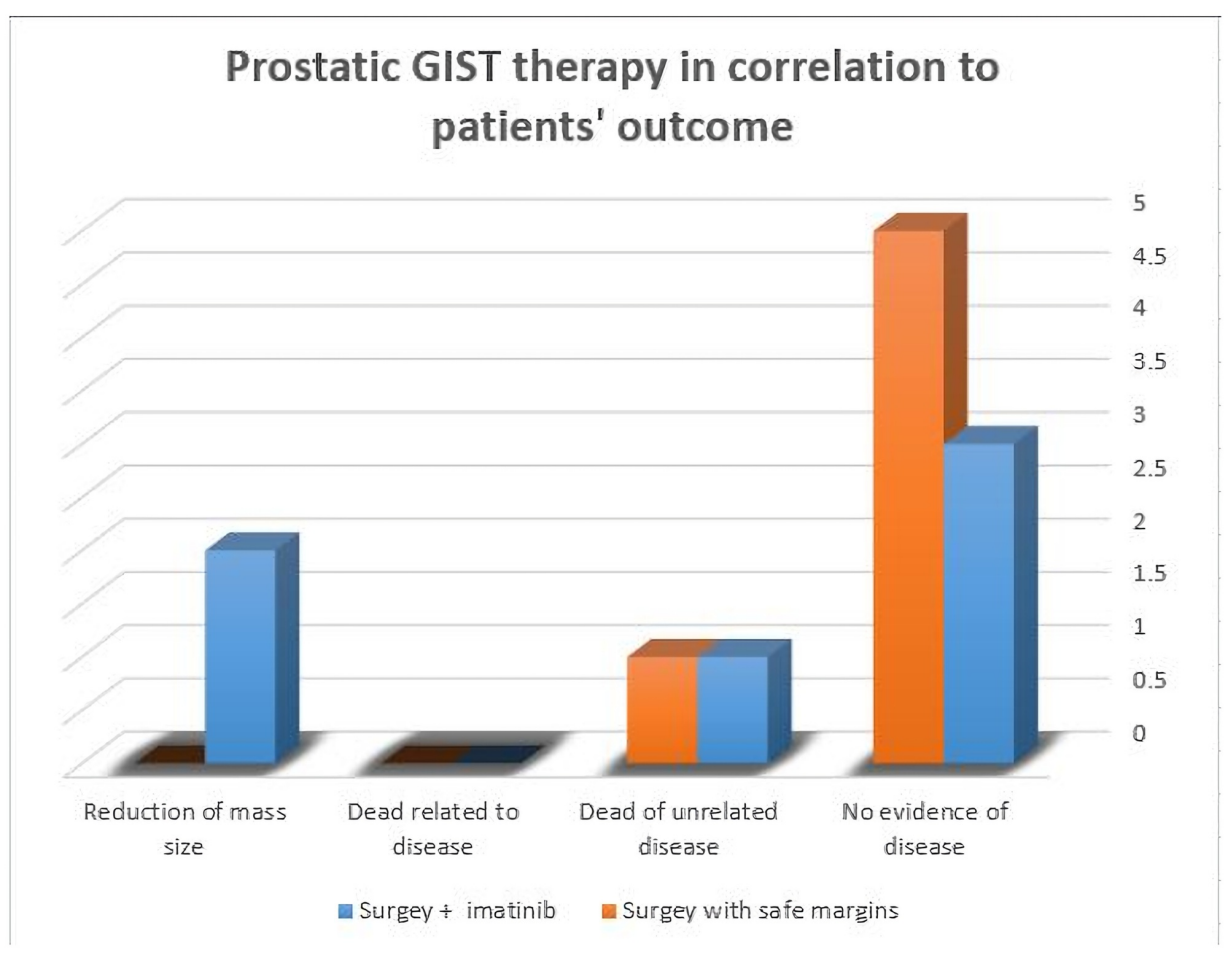
A bar chart demonstrating primary prostatic GIST and patient outcome GIST: Gastrointestinal stromal tumor

GISTs can have hidden potential for recurrence and distant metastasis many years after the initial excision. They respond to imatinib mesylate (Gleevec) and sunitinib malate targeted therapies. Both reagents demonstrated an effective response approved by the US Food and Drug Administration (FDA) [24]. Mutation status analysis for each GIST case can be of great help for oncologists; this can enhance the impact of which drug is suitable to be used. A tumor with treatment changes effect can show tumor fibrosis, hypocellularity, myxoid changes, and/or necrosis. We believe it is helpful to mention the percentage of viable tumor cells after therapy [2]. Surgical intervention with clear margins is a very effective treatment modality for resectable tumors. However, cases with a large unresectable mass, high-grade, high recurrence rate, or metastasis can be treated with imatinib. Cases with potentially resectable tumors are preferred to use neoadjuvant imatinib for tumor size reduction, which significantly decreases surgical morbidity. Yet patient survival and neoadjuvant imatinib is not properly studied. Figure 3 manifests a bar chart demonstrating the treatment methods used for previously reported cases of primary prostatic GIST and their patient's outcome.

Discussion

The diagnosis of extra-gastrointestinal GIST (E-GIST) was established when reported cases of GIST were seen primarily outside the GI tract such as bowel mesentery, omentum, retroperitoneum, and pleura [25]. Primary visceral GIST is extremely rare and not well studied in the literature. Organs such as the liver and spleen mostly represent secondary involvement by GIST from distant GI metastasis or occult primary GI. Interestingly, some E-GISTs that arise in the GI tract primarily can get detached substantially from their primary GI site, which can be discovered lately as an occult tumor site [22]. Due to the lack of cell of Cajal in the E-GIST site, some pathogenesis theories proposed that it can arise from ectopic interstitial cells of Cajal. These cells were originated from the pluripotential progenitor of mesenchymal cells. Tumor pathogenesis, morphology, and molecular alterations of E-GIST are similar to classical GIST of the GI tract. However, E-GISTs of the mesentery and retroperitoneum are more clinically aggressive than cases that arise in the omentum [26]. An extensive literature review reveals many studies and reported cases of GIST diagnosed in a core needle biopsy of prostate tissue. However, there must be a clear index and convincing clinical evidence to claim it is a primary prostatic GIST tumor. The prostate gland is located in a unique anatomical location, which is more accessible for tissue sampling. This can make secondary involvement by an adjacent organ highly possible. GIST diagnosed in a prostatic biopsy can be from a GIST tumor arising primarily in the rectum and sampled on prostate needle tissue, or a sampled tissue from GIST located between the rectum and prostate, or GIST arising primarily in the prostate gland.

A GIST must be considered in the differential diagnosis of mesenchymal lesions of the prostate along with appropriate immunohistochemistry studies. The correct diagnosis of GIST will affect the clinical management of the patient, as tumors respond to tyrosine kinase inhibitor STI571. The KIT protein is a tyrosine kinase receptor molecule expressed normally in plenty of cells such as the cells of Cajal, germ cells, and mast cells [27]. The KIT-activating mutation usually occurs at exon 11, rarely at exons 9 and 13, and is usually absent in pediatric and syndromes-associated GIST. The detection of mutation by immunohistochemistry studies can be achieved as a result of ligand-independent receptors activation. CD117 (c-KIT) provides a useful membranous and some cytoplasmic staining, unlike other mesenchymal tumors, which stain only cytoplasmic pattern with coarse granules [28]. However, rare cases of GIST can be negative for CD117 and, yet, a KIT mutation is detected. CD117 (c-KIT)-negative GISTs are usually non-conventional, have an epithelioid morphology, a myxoid stromal background, abundant mast cells, and are commonly located in E-GIST locations, such as the omentum, which harbors PDGF-a receptors mutation [29]. DOG1, which stands for Discovered On GIST-1, is a very sensitive and specific marker for GIST, which stains interstitial cells of Cajal in the GI tract [30]. It is sensitive for CD117-negative GIST, and E-GIST is usually highlighted by DOG1 in a membranous and cytoplasmic staining pattern [31]. Some authors suggest that if DOG1 staining is negative, it is not a GIST [32].

The differential diagnosis includes tumors related to prostatic tissue such as prostatic stromal tumors of uncertain malignant potential (STUMP), sclerosing adenosis, prostatic stromal sarcoma, and sarcomatoid carcinoma. Other infrequent differentials are solitary fibrous tumor (SFT), IMT, synovial sarcoma, rhabdomyosarcoma, and smooth muscle tumors such as leiomyoma and leiomyosarcoma. Angiosarcoma, haemangioma, and granular cell tumors should be excluded [33]. STUMP can present with different histological patterns, all of them can have degenerative nuclear changes and smudged nuclear chromatin. The first pattern is hypercellular stroma with degenerative nuclear atypia and vacuolated nuclei, the second is hypercellular stoma with bland cellular changes, the third is a "leaf-like" pattern mimicking benign phyllodes tumor of the breast with hypocellular fibrotic stromal changes, and the fourth pattern is myxoid stromal changes with spindle cells proliferation lacking benign nodular hyperplasia of the prostate [34]. STUMP can have an epithelial component in the form of crowding of glands, basal cell hyperplasia, and infoldings of papillary projections, which can be tricky in needle biopsy and may mimic adenocarcinoma. STUMP can exhibit a mixture of the mentioned patterns. Mitosis is rare or even absent and necrosis is not present. STUMP is positive for CD34 (which can be expressed in GIST) and variably positive for SMA, Desmin, and Actin, muscle-specific antibody (HHF-35) while negative for CD117 and DOG1. Unlike sarcomatoid carcinoma, STUMP is negative for cytokeratin staining. STUMP shows no correlation between its different morphological patterns and association with stromal sarcoma. Sclerosing adenosis of the prostate is a benign lesion that can have a prominent spindle cell stromal proliferation. In a core tissue biopsy, diagnosis can be challenging especially when epithelial component proliferation is minimal or not enough sample. The spindle cell component of these lesions lacks nuclear atypia. These spindle cell elements are believed to be originated from a myoepithelial linage of the epithelial components and that may explain the positive staining for high-molecular-weight cytokeratin (HMWCK). They are negative for GIST immune markers. Prostatic stromal sarcoma is defined as one or more of the following: hypercellularity, cytological atypia, mitotic figures, and necrosis. It tends to affect the younger age population, where most of the reported cases were younger than 50 years old. Stromal sarcoma histological patterns can be storiform, fibrotic, epithelioid, or sheet of cells infiltrating between remaining benign prostatic glands. A leaf-like pattern with hypercellular stroma is less likely found in stromal sarcoma. Immunohistochemical studies are like STUMP, with strong vimentin staining and positive CD34. Pancytokeratin, CAM5.2, CD117, and DOG1 are negative. Sarcomatoid carcinoma (carcinosarcoma) is usually found in patients with a previous history of adenocarcinoma of the prostate. Histopathology can be a variable between a mixture of malignant epithelial and mesenchymal components. The mesenchymal component can reveal clear features of malignancy such as stromal hypercellularity, large hyperchromatic nuclei, abundant mitoses, tumor necrosis, and bizarre giant tumor cells. Heterologous elements can aid the diagnosis of carcinosarcomas such as osteosarcoma, chondrosarcoma, or rhabdomyosarcoma components [33]. SFT commonly affects the pleura; however, it has been described in other vesical organs, including the genitourinary system. Prostatic SFT was reported in the age group ranging from 22-75 years old, with a size range between 2 cm and 14 cm. SFT classically forms the “staghorn” pattern of thin-walled branching blood vessels characteristically seen in tumors with a hemangiopericytoma pattern. This is a very useful diagnostic clue in resection specimens, but it can be very difficult to appreciate in biopsy tissue samples. The neoplastic cells are uniform bland spindle cells, and they are frequently seen in a variable background of ropy collagen. SFT lesions usually do not have an admixed, entrapped prostatic tissue. Stromal myxoid changes can be seen, which can mimic GIST, however, the overall architecture and morphology of bland elongated cells with minimal cytoplasm, small nuclei, indistinct nucleoli, and perivascular sclerosis can favor SFT. Malignant SFT has more cellular overgrowth, pleomorphism, a high nuclear to cytoplasmic ratio, tumor necrosis, mitotic figures more than four per 10 high power field (HPF), and irregular infiltrative margins. Further immunohistochemistry is necessary for diagnosis confirmation. Most important is nuclear staining for signal transducer and activator of transcription 6 (STAT6). It is also positive for other nonspecific markers like CD34, CD99, B-cell lymphoma 2 (BCL-2), and vimentin and negative for pan-cytokeratin, S-100, CD117, and DOG1 [35]. IMT commonly arises in the urinary bladder, which is composed of bland cell proliferation of spindled myoepithelial cells with lymphocytic infiltrate. Stellate myofibroblasts with abundant eosinophilic cytoplasm are clues to the diagnosis. IMTs are positive for SMA, Desmin, and anaplastic lymphoma kinase (ALK1) (75%-89% of cases). They usually have ALK gene rearrangements by Fluorescence In Situ Hybridization (FISH) [36]. Synovial sarcoma can occur anywhere in the body. It can be difficult to diagnose it based on needle tissue biopsy especially if it is a monophasic spindle cell type. A histological clue to the diagnosis is hypercellular fascicular cellular architecture with little intervening stroma in between. Their nuclei often close enough to overlap with adjacent cell nuclei. Mast cells and focal calcification can be seen. Transducin-like enhancer of split 1 (TLE1) is positive in 80%-90% of cases [37]. Epithelial markers such as cytokeratin and Epithelial membrane antigen (EMA) are positive but negative for CD34, Desmin, h-Caldesmon, myogenin, myoblast determination protein 1 (MyoD1), CD117, and DOG1 [38].

Rhabdomyosarcomas are primitive mesenchymal tumor cells that show many degrees of immature skeletal muscle differentiation. They can have both the morphology of hypo- and hypercellular areas along with loose myxoid stromal changes, mimicking the GIST background. However, it is critical to document positive staining for Desmin, MyoD1, and myogenin. While negative for DOG1, CD117, and CD34 [39]. Finally, smooth muscle tumors, such as leiomyoma and leiomyosarcoma, are composed of smooth muscle bundles proliferation. Leiomyoma are composed of elongated cells with eosinophilic cytoplasm and distinct cellular membrane. The diagnosis of leiomyosarcoma requires two out of three histologic criteria (marked cellular atypia, more than 10 mitoses/10 HPF, and tumor necrosis). They are positive for smooth muscle markers, including SMA, Caldesmon, and Desmin, while negative for DOG1, CD117, and CD34 [40].

## Conclusions

GIST should be always considered in the differential diagnosis of prostatic spindle cell lesions. Due to the therapeutic impact, CD117 immune staining must be always investigated and included in the diagnostic panel. Surgical resection with clear margins is considered the main treatment in large bulky masses. Early GISTs respond well to imatinib therapy along with surgical resection if needed. The duration of therapy has no impact on the tumor recurrence rate, but it affects the overall patient survival rate.
